# Pubertal development in healthy children is mirrored by DNA methylation patterns in peripheral blood

**DOI:** 10.1038/srep28657

**Published:** 2016-06-28

**Authors:** Kristian Almstrup, Marie Lindhardt Johansen, Alexander S. Busch, Casper P. Hagen, John E. Nielsen, Jørgen Holm Petersen, Anders Juul

**Affiliations:** 1 Department of Growth and Reproduction, Section GR-5064, Rigshospitalet, University of Copenhagen, Blegdamsvej 9, DK-2100 Copenhagen, Denmark; 2 International Center for Research and Research Training in Endocrine Disruption of Male Reproduction and Child Health (EDMaRC)Copenhagen, Denmark.; 3 Institute of Public Health, Department of Biostatistics, University of Copenhagen, Copenhagen, Denmark

## Abstract

Puberty marks numerous physiological processes which are initiated by central activation of the hypothalamic–pituitary–gonadal axis, followed by development of secondary sexual characteristics. To a large extent, pubertal timing is heritable, but current knowledge of genetic polymorphisms only explains few months in the large inter-individual variation in the timing of puberty. We have analysed longitudinal genome-wide changes in DNA methylation in peripheral blood samples (n = 102) obtained from 51 healthy children before and after pubertal onset. We show that changes in single methylation sites are tightly associated with physiological pubertal transition and altered reproductive hormone levels. These methylation sites cluster in and around genes enriched for biological functions related to pubertal development. Importantly, we identified that methylation of the genomic region containing the promoter of *TRIP6* was co-ordinately regulated as a function of pubertal development. In accordance, immunohistochemistry identified TRIP6 in adult, but not pre-pubertal, testicular Leydig cells and circulating TRIP6 levels doubled during puberty. Using elastic net prediction models, methylation patterns predicted pubertal development more accurately than chronological age. We demonstrate for the first time that pubertal attainment of secondary sexual characteristics is mirrored by changes in DNA methylation patterns in peripheral blood. Thus, modulations of the epigenome seem involved in regulation of the individual pubertal timing.

Puberty is associated with the development of secondary sexual characteristics and attainment of adult reproductive capacity. This transition from child to adult phenotype is initiated by central activation of the hypothalamic–pituitary–gonadal (HPG) axis resulting in stimulation of steroid hormone synthesis. The ages at which puberty starts vary markedly both among healthy boys (9–14 years) and girls (8–13 years) and is to a large extent (60%) heritable^1^. Candidate gene approaches have identified single genes associated with pubertal extremes like precocious puberty^2^ or absence of puberty^3^. However, no single gene appears to account for the large variation in pubertal timing among children with normally timed puberty. In fact, recent genome-wide association studies explain very little of this normal variation^4^,^5^, and the largest genetic effect on pubertal onset has been found by a candidate gene approach within *FSHR* and *FSHB*^6^. Furthermore, the secular trend of declining age at pubertal onset in both sexes in the western world has been observed over just a few decades, and must therefore be attributed to other factors than genetic variation.

Profiling of DNA methylation patterns (methylation of cytosine in the context of a CpG site) in lymphocytes from human peripheral blood samples has been used to predict physiological outcomes or prospectively associate patterns with events earlier in life. Some of the most successful studies include prediction of biological aging^7^,^8^,^9^,^10^,^11^,^12^, associations with smoking^13^,^14^, and obesity^15^,^16^ as well as persistent changes in adults following *in utero* famine during the second world war^17^,^18^. A link between self-reported menarcheal age and peripheral blood DNA methylation patterns has previously been investigated^19^ but the results were rather inconclusive due to study design and technical aspects.

In this longitudinal study of healthy Danish boys and girls with repeated blood sampling, we investigated the relationship between individual changes in genome-wide DNA methylation patterns and pubertal development. We identified several CpGs and CpG islands that closely followed pubertal transition suggesting important novel regulators of pubertal onset.

## Results

**Figure 1:**
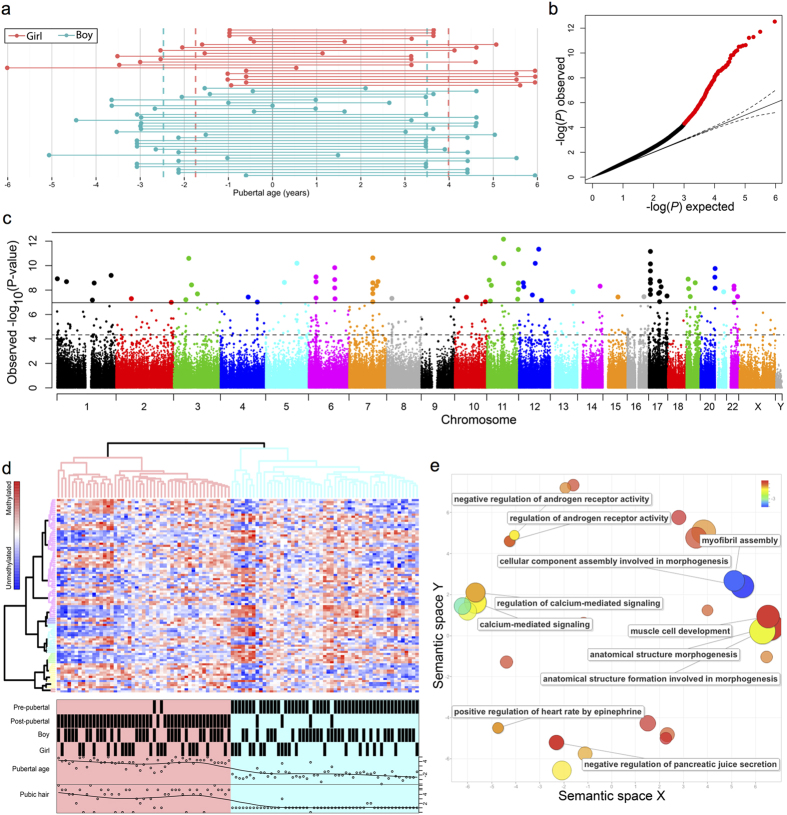
Identification of CpGs associated with pubertal age. (**a**) From the COPENHAGEN puberty study of children followed longitudinally, selected pre- and post-pubertal samples from 31 boys and 20 girls were included in the study. Time relative to onset of puberty as measured by a testicular volume of 4 ml or breast tanner stage B2 or above was calculated and defined as the pubertal age. (**b**) Genomic inflation of the genome-wide DNA methylation patterns correlating with pubertal age were corrected by applying an improved surrogate variant analysis (SmartSVA) resulting in a genomic inflation factor of 1.2 and a qq-plot leaving 457 significant CpG sites at a FDR of 0.05 (red). (**c**) Manhattan plot of the significant CpG sites reveal a distribution across all autosomes. (**d**) Unsupervised hierarchical centroid clustering of CpGs associated with pubertal age. The resulting dendrograms were colour coded according to their height and divided samples into two major groups that nearly uniquely represented pre- and post-pubertal boys and girls. (**e**) Gene ontology analysis of all significant CpGs revealed several ontologies (p-value < 0.05) that could be related to pubertal development. Biological process gene ontologies were plotted in a sematic space, using REVIGO, that groups related ontologies together.

From our longitudinal cohort (the COPENHAGEN puberty cohort) of healthy boys and girls, followed clinically during pubertal development, we included 22 girls and 32 boys who all had blood samples drawn for DNA isolation before and after onset of puberty. Pubertal onset was identified by thorough clinical investigation and defined as a testicular volume of ≥4 ml, or Tanner breast stage ≥B2 for boys and girls, respectively. The mean age between two examinations where this threshold was reached was used as their age of pubertal onset. Pre-pubertal blood samples were drawn at a mean age of 9.1 and 8.3 years corresponding to −2.5 and −1.8 years prior to pubertal onset for boys and girls, respectively. Post-pubertal samples were drawn at a mean age of 15.1 and 14 years and 3.5 and 4 years after pubertal onset for boys and girls, respectively (Fig. 1a, Table 1). Two girls and one boy were excluded for technical reasons. We observed a slight tendency towards lower DNA methylation levels in open sea and in shores and shelf of CpG islands when pre-pubertal were compared to post-pubertal children and significantly (p = 0.001) higher DNA methylation levels of CpG islands in girls compared to boys (Supplementary Figure 1).

### Identification of differentially methylated CpGs associated with pubertal timing

Each time point for DNA collection was assigned a specific pubertal age (i.e. years from pubertal onset). In this way, we identified CpGs that were associated specifically with pubertal development. A modified surrogate variant analysis (SmartSVA) was applied to correct for confounding effects (e.g. possible differences in blood composition) and reduced the genomic inflation to 1.2 (Fig. 1b). When boys and girls were analysed together (corrected for age and sex) 457 CpGs with a false discovery rate (FDR) below 0.05 were identified. They were evenly distributed across all autosomes (Fig. 1c and Supplementary Table 1). Unsupervised hierarchical clustering of the most significant CpGs (94 CpGs at FDR < 0.001) resulted in nearly complete separation of children according to whether samples were obtained in pre-pubertal vs. post-pubertal states (Fig. 1d). When boys and girls were analysed separately, only the data from the boys resulted in significant CpGs (data not shown).

The core list of the 94 CpGs associated with pubertal age was used to search for significantly enriched gene ontology terms (biological process, p < 0.05) and subsequently plotted in a semantic space that group similar terms (Fig. 1e). This revealed clusters related to e.g. regulation of androgen receptor activity, anatomical morphogenesis, muscle cell development, and pancreatic juice secretion (Supplementary Tables 2 and 3).

### Differentially methylated CpGs associated with circulating reproductive hormone levels

**Figure 2:**
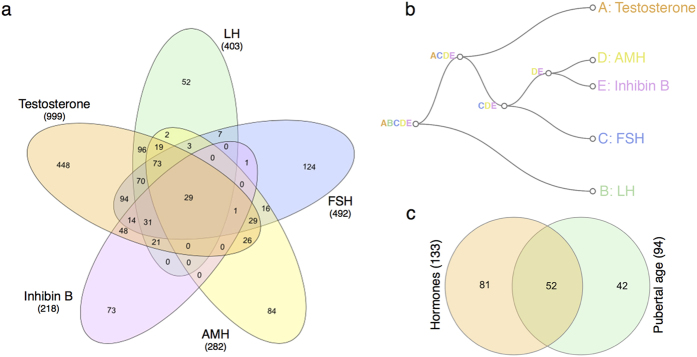
Identification of CpGs associated with changes in circulating reproductive hormones in boys. (**a**) Venn diagram of CpGs associated with Testosterone (n = 999), FSH (n = 492), LH (n = 403), AMH (n = 282), and Inhibin B (n = 218) at a FDR cut-off of 0.05 in boys after correcting for genomic inflation and age. (**b**) Binary tree obtained from a sequence of union operations showing how related the hormone CpG clusters are to each other. (**c**) Venn diagram of the CpGs associated with pubertal age and circulating reproductive hormones (Supplementary Tables 1, 5 and 6).

Pubertal transition entails central activation of the HPG axis and hence increases in circulating reproductive hormones; a process which is markedly different between boys and girls. We therefore compared changes in DNA methylation to changes in reproductive hormones independently in boys and girls. Using a similar SmartSVA method as applied for pubertal age (above) to control for genomic inflation, a FDR cut-off of 0.05 identified no significant CpGs among the girls (that start to cycle post-pubertally). Among the boys, we identified 999, 492, 403, 282, and 218 CpGs that were associated with changes in testosterone, follicle-stimulating hormone (FSH), luteinizing hormone (LH), anti-Müllerian hormone (AMH), and Inhibin B, respectively (Supplementary Table 4, Fig. 2a). A binary tree obtained from a sequence of union operations between the CpGs revealed that LH CpGs were more distant than the others and that Inhibin B, AMH, and FSH were more related (Fig. 2b). As for pubertal age, a core subset of 133 CpGs associated with at least 3 of the 5 reproductive hormones (Supplementary Table 5) separated children into pre- and post-pubertal in an unsupervised hierarchical clustering (Supplementary Figure 2). Importantly, the core sets originating from CpGs associated with pubertal age and reproductive hormones, respectively, showed a highly significant overlap (p-value: < 0.0001; Fig. 2c). 52 CpGs showed association to both reproductive hormones and pubertal age (Supplementary Table 6) and included 5 (10%) and 6 (12%) CpGs in the proximity of the genes *TRIP6* and *KCNAB3*, respectively.

### Genomic regions with differential methylation

**Figure 3:**
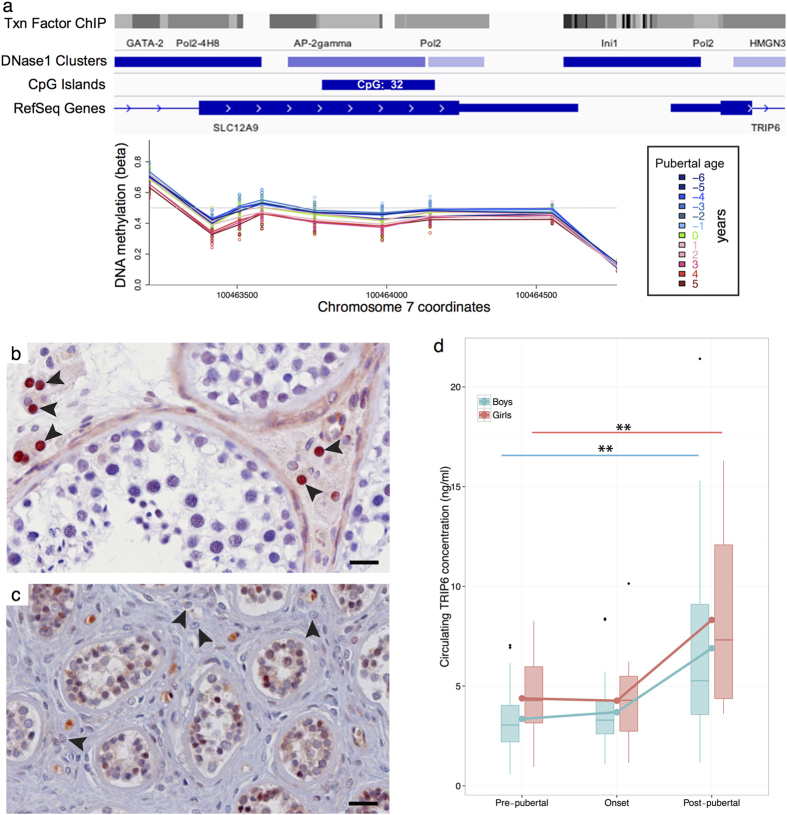
Identification of differentially methylated regions associated with pubertal age. (**a**) The most significant differentially methylated genomic region (nine probes with a mean p-value of 1.3e–31) was found on chromosome 7 (chr7:100463206-100464771) that contains both gene body and 3′ UTR of *SLC12A9* and transcription start site of *TRIP6*. The CpGs were located in several putative transcription factor bindings sites upstream of *TRIP6*. TX Factor ChIP, DNase1 clusters tracks were obtained from the ENCODE database and the CpG Islands from the UCSC database. (**b**) Immunohistochemical staining for TRIP6 in normal adult testicular tissue showing intense and specific staining in Leydig cells (arrow heads) producing testosterone. (**c**) TRIP6 staining of pre-pubertal (7.9 years old) testicular tissue. TRIP6 staining was absent from Leydig cells (arrow heads) in pre-pubertal testis. Bar equals 20 μm. Negative controls are shown in Supplementary Figure 4 together with staining of ovarian tissue. (**d**) Circulating levels of TRIP6 were determined by ELISA in boys and girls pre-pubertally, around pubertal onset and post-pubertally. The box plots show the distribution of the measurements (the band inside the box depicts the median) at each time point and the connected lines are drawn from the mean of each group. **Denotes a p-value below 0.01.

We searched for genomic regions (in a window of 1000 bases) where DNA methylation in a co-ordinated manner was associated with pubertal age. This identified genomic regions that were differentially methylated in boys and girls individually (data not shown) and in both genders collectively (Supplementary Table 7). The most significant region for both genders, when analysed individually (Supplementary Figure 3) as well as collectively (Fig. 3a), was a region on chromosome 7 situated between *SLC12A9* and *TRIP6* (hg19 coordinates chr7:100463206-100464771). This region contains the last exon and 3′ UTR of *SLC12A9* and the promoter of *TRIP6.* It furthermore contains both a CpG island and several potential binding sites for transcription factors (Fig. 3a). Since the region was upstream of *TRIP6* and included several potential transcription factor binding sites, we analysed the expression of TRIP6 by immunohistochemistry in a target tissue for pubertal transition, i.e. testicular tissue from healthy pre- and post-pubertal (adult) males. This revealed a prominent and highly specific expression in adult testosterone producing Leydig cells (Fig. 3b), whereas TRIP6 expression in pre-pubertal Leydig cells was absent (Fig. 3c). TRIP6 staining was also found in the less mature Sertoli cells in the pre-pubertal sample but not in the mature adult testis. Ovarian tissue showed a weak staining in oocytes and granulosa cells, whereas the theca cells were negative for TRIP6 staining (Supplementary Figure 4). In addition to expression in target tissues, we also analysed the circulating levels of TRIP6 in a subset of pre-, mid- (around onset) and post-pubertal children (20 boys and 18 girls). This showed a significant increase in the circulating levels as children progressed through puberty (Fig. 3d). TRIP6 levels doubled in boys from 3.4 (95% CI: 2.5–4.2) ng/ml pre-pubertally to 6.9 (4.5–9.3) ng/ml post-pubertally. Even though variation was observed, the increase was highly significant (p-value: 0.005). Similar data was obtained in girls where the mean pre-pubertal levels were 4.4 (3.4–5.4) ng/ml and significantly increased (p-value: 0.004) to 8.3 (6.1–10.5) ng/ml in post-pubertal girls. In general, TRIP6 levels appeared to be higher in girls than in boys, and the *TRIP6* promoter also seemed to be more differentially regulated in girls (Fig. 3d and Supplementary Figure 3).

Moreover, changes in methylation levels of the same genomic region between *SLC12A9* and *TRIP6* were significantly associated with testosterone (boys), FSH (boys and girls), and LH (boys and girls) levels. This was however not the case for Inhibin B (boys and girls), AMH (boys and girls), or estradiol (girls; data not shown).

In addition, a region in the proximity of *KCNAB3* was identified as the third most significant region (Supplementary Table 7) to be differentially methylated during pubertal transition.

### Prediction of pubertal age from DNA methylation patterns

**Figure 4:**
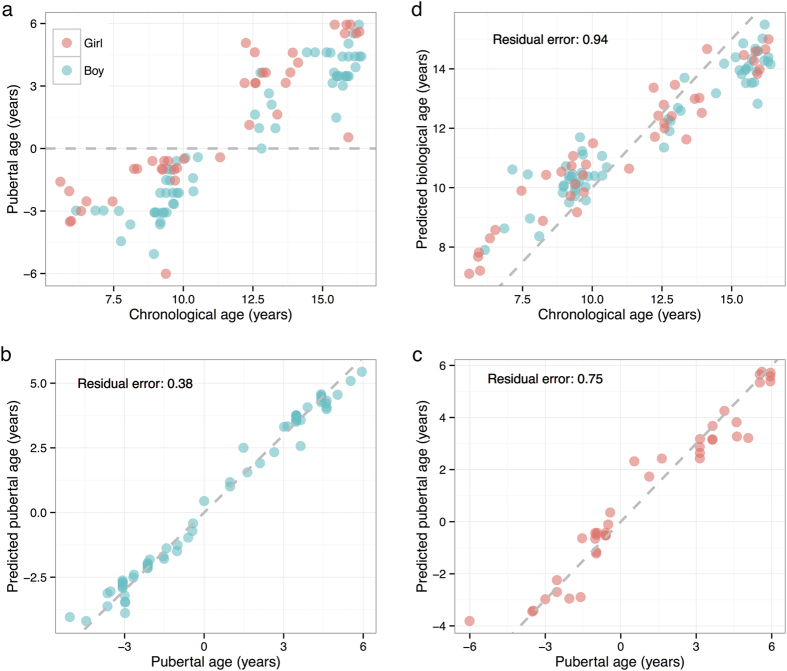
Prediction models of pubertal and biological aging. (**a**) Scatter plot of chronological age and pubertal age. Using an elastic net prediction model and a 10-fold cross validation, the pubertal age was predicted from DNA methylation patterns in peripheral blood from (**b**) boys and (**c**) girls. A residual error of 0.39 and 0.74 year, equalling five and nine months was observed in boys and girls, respectively. (**d**) Prediction of biological aging from our data using the same algorithm as applied for pubertal age revealed a residual error of 0.94 year (11 months). The dashed red line is the identity line.

DNA methylation patterns have been shown to predict biological aging, and with our longitudinal data on pubertal age (Fig. 4a), we therefore tried to predict the pubertal age of boys and girls based on our methylation data. Normalised and filtered probes (n = 467,873) were used as predictors in an elastic net regression model (similar to the algorithms earlier used in prediction of biological aging). The prediction error (standard deviation of residual error) was estimated using 10-fold cross validation and corrected for chronological age. Using the minimal lambda penalisation value (0.10 and 0.13), the pubertal age was predicted with a residual error of 0.39 and 0.74 year, equalling five and nine months in boys and girls, respectively (Fig. 4b,c). In comparison, the residual error obtained from our data on predicted biological aging (Fig. 4d) was 0.94 year (11 months; lambda = 0.06). When boys and girls were combined into one analysis, the residual error was 0.6 year (seven months) for prediction of pubertal age.

## Discussion

To our knowledge, this is the first human longitudinal study describing individual changes in specific epigenetic profiles associated with pubertal onset. We identified a number of CpGs and CPG islands, suggesting novel key players for pubertal development.

Our study cannot reveal the cause and effect of differentially regulated CPGs. However, a downstream biological consequence of a change in a single CpG is less likely and, as such, these may represent surrogate markers of pubertal transition. It is more likely that coordinated changes of DNA methylation in genomic regions have a downstream biological effect. Consequently, we searched for such regions in our data and identified a potential regulatory region upstream of *TRIP6* that was co-ordinately regulated during pubertal transition. Not much is known about human TRIP6 (Thyroid Hormone Receptor Interactor 6, 7q22), which according to ClinVar ( http://www.ncbi.nlm.nih.gov/clinvar/; RRID:SCR_006169) does not contain any SNPs that are clearly associated to a clinical phenotype and no *TRIP6* variants were identified in recent large association studies on age at menarche^4^,^20^. It contains three LIM zinc-binding domains and interacts with TR-beta only in the presence of thyroid hormone^21^. As a member of the zyxin family, TRIP6 has also been described to be involved in actin cytoskeleton rearrangements and was recently shown to be required for F-actin organization and dendritic morphology of hippocampal neurons^22^. TRIP6 is also involved in nuclear factor kappa B and JNK signalling by LPA2 receptor and TRAF6 binding^23^ and modulates glucocorticoid receptor transcriptional activity^24^. According to FANTOM5 (RRID:SCR_002678) data, *TRIP6* is found expressed in many tissues but is, in particular, found in reproductive tissues. Detailed investigation of gene expression profiles in the GEO database (RRID:SCR_004584) provides evidence of differential *TRIP6* expression during rodent development of the mammary gland (GDS2721 and GDS2360)^25^,^26^, gonads (GDS4503)^27^, and hypothalamus (GDS2862) as well as in the hypothalamic hamartomas of patients with central precocious puberty (GDS3110)^28^. Furthermore, we found TRIP6 to be highly and specifically expressed in human testicular Leydig cells (producing testosterone) in healthy adult men but not in pre-pubertal boys. In contrast to mature Sertoli cells, TRIP6 was expressed in immature Sertoli cells in pre-pubertal testes. It is well known that thyroid hormones play an important role in regulation of both brain and testicular development. In the testis, the most potent thyroid hormone, triiodothyronine (T3), induce androgen receptor expression in immature Sertoli cells^29^ that together with FSH drives Sertoli cell proliferation and consequently induction of spermatogenesis during onset of puberty. Importantly, T3 also induces both StAR and LHR expression in Leydig cells^30^ and T3 therefore seems to be a key player in the pubertal transition of the testis. We speculate that TRIP6 may be involved in the T3 activation of steroidogenesis in Leydig cells during pubertal onset in boys, which is reflected by promoter demethylation of *TRIP6* in white blood cells. Similarly in females, T3 increases FSH-induced antral follicle growth *in vitro* and regulates granulosa cell proliferation^31^,^32^. Doubling of circulating levels of TRIP6 in pre- to post-pubertal children even further substantiates that TRIP6 is induced during pubertal transition. To our knowledge, only the closely related zyxin-family members *zyxin* and *Lpp*, and not *Trip6,* have been knocked out in mice^33^,^34^. Drosophila contains only one *TRIP6* orthologue, zyxin, and null mutants yields smaller flies, which however could be related to an effect on actin rearrangements^35^.

Another interesting, and nearly as significant region as the *TRIP6* promoter, was found in front of *KCNAB3* (Voltage-gated potassium channel subunit beta-3). However, very little is currently known about KCNAB3, which very interestingly, is thought to function primarily in the brain^36^,^37^. Mice lacking *Kcnab3* weigh less than control littermates probably due to a higher basal metabolic rates but it is unknown whether pubertal development was affected^38^.

Many parameters have been shown to influence DNA methylation in peripheral blood. Some of the identified pubertal CpGs could potentially be changed due to e.g. biological aging or changes in the cellular composition of blood. However, the age at pubertal onset varied substantially and importantly also the age of the children when blood samples were drawn (5.6–11.3 and 12.2–16.4 years of age, pre- and post-pubertally, respectively). Moreover, the regression analysis was adjusted for age at blood sampling by including it as a non-penalized covariate in the prediction model. Similarly, boys and girls entered puberty at different ages and sex. Consequently, sex was also included as a specific covariate. We tried to analyse boys and girls separately, and the overall picture was that only significant CpGs could be identified among the boys. We speculate that this is partly due to the lower number (and hence lower power) of girls (20 girls and 40 samples) included in the analysis compared to boys (31 boys and 62 samples), but also due to reproductive hormone cyclicity in post-pubertal girls. It is evident that e.g. differential methylation of the *TRIP6* promoter is regulated also in girls when analysed alone (Supplementary Figure 3), indicating that similar pubertal methylation profiles are found in both sexes. A significant proportion (34 out of 94), but not all, of the CpGs associated with pubertal age in boys and girls were also identified when boys were analysed alone, indicating added statistical power by inclusion of girls in the analysis. Moreover, it should also be considered that the Tanner staging of boys and girls may not resemble the same pubertal readout and the same “absolute stage” (breast tissue vs. testicular volume). Altogether, our results indicate that future studies should attempt to include a larger amount of children, which should provide statistical power to analyse boys and girls separately. Isolation of specific subsets of lymphocytes may in addition provide further power to the analysis. In general we observed higher methylation levels of CpG islands in girls compared to boys, which is in accordance to earlier reports on sex differences of overall DNA methylation levels^39^.

We did not have cell counts on the different lymphocyte populations in the samples, but we believe that this potential bias was efficiently corrected for by applying the SmartSVA method. The SmartSVA method was, in our study, superior to the EWASher method as EWASher, besides reducing the genomic inflation, also reduced the significant signals substantially. As white blood cells cannot be regarded as a target tissue for pubertal development, we speculate that the identified changes in epigenetic profiles associated with pubertal transition may be traces of the regulation that takes place in target tissues. Indeed, the gene ontology analysis identified many terms that would normally be regarded as processes taking place during pubertal transition in target tissues. In particular, terms related to morphogenesis and HPG activation were identified. Recently, *Finucane et al.*^40^ reported gene ontology analysis of GWAS data on “age at menarche” and found enrichments of “Adrenal or pancreas” and “CNS”, which fit very well with the terms found in our gene ontology analysis. A link between epigenetic events in blood cells and epigenetic regulation in reproductive target tissue is further substantiated by the above-mentioned changes in *TRIP6* promoter methylation and coordinated changes in expression in Leydig cells during pubertal transition as well as circulating TRIP6 levels.

Many studies have investigated the relationship between DNA methylation patterns and biological aging^7^,^8^,^9^,^10^,^11^,^12^, which currently is the best predictor of chronological age. It has been reported that overall DNA methylation levels decrease by age^41^, which is the same tendency observed in our data. Individuals that show a progressed biological aging compared to their chronological age have a greater risk of all-cause mortality^11^. Furthermore, cancer tissues show accelerated biological aging^8^. In our study, we were able to predict pubertal age even better than biological aging. Many factors including the paired design, the age intervals investigated and other technical issues could influence the performance of the prediction accuracy, but our results clearly indicate that it is possible to accurately predict the pubertal development independently of age with a good accuracy. It would be interesting to study children with precocious or delayed puberty to investigate whether these conditions are reflected in DNA methylation patterns. Likewise, it would be intriguing to investigate patients with altered reproductive hormone levels. The described tight link between pubertal development and DNA methylation may, however, also imply that disrupting environmental factors affecting the pubertal methylome could be involved in the declining age at pubertal onset observed in the western world.

In summary, pubertal transition was reflected by changes in the patterns of DNA methylation in peripheral white blood cells. Changes in methylation of single CpGs were associated to both the stage of pubertal development (pubertal age) as well as changes in circulating hormones and could be used to predict the pubertal age. Methylation levels of genomic regions, including the promoter of *TRIP6,* systematically changed during pubertal transition. Epigenetic activation of *TRIP6* was reflected by post-pubertal expression in reproductive target tissue as well as increasing circulating levels. Our human data is the first to indicate that peripheral blood is a valuable surrogate tissue for assessment of sexual maturity during pubertal transition.

## Methods

### Study population and clinical examination

The COPENHAGEN Puberty Study (ClinicalTrials.gov ID: NCT01411527) is a combined cross sectional and longitudinal population-based cohort study of healthy Danish children and adolescents. The study population has been described in detail previously^42^,^43^,^44^ and has been approved by the local Danish ethical committee (KF 01 282214; V200.1996/90) and the Danish Data Protection Agency (2010-41-5042). The study was carried out in accordance with the approved guidelines and written informed consent was obtained from all children and parents. In this study, we included 32 boys and 22 girls. The clinical evaluations were performed by trained physicians and included pubertal staging of breast development according to Tanner’s classification evaluated by palpation^45^. Testicular volume was determined using orchidometry as described by Marshall and Tanner^45^. A breast Tanner stage of two or above and a testicular volume of 4 mL or more were considered to be markers of pubertal onset in girls and boys, respectively. The age of pubertal onset was approximated using the date exactly between two visits where the girls advanced from B1 to B2 (or more) and the boys’ testicular volume increased to 4 ml or above. In this study, we used the estimated date of onset to determine the pubertal age, defined as the difference between the date of examination relative to the age at onset.

Serum levels of FSH and LH were measured by a two-sided time-resolved fluoroimmunoassay (Delfia, Wallac, Inc., Turku, Finland) with detection limits of 0.06 and 0.05 IU/L, respectively. Total serum estradiol was measured by a RIA (Pantex Corp., Immunodiagnostic Systems Limited, Santa Monica, CA, USA) with a detection limit of 18 pmol/l. Serum AMH levels were determined using the Beckman Coulter enzyme immunometric assay generation I (Immunotech, Beckman Coulter Ltd., USA) with a detection limit of 2.0 pmol/L. Serum inhibin B was measured using a double antibody immunometric assay (Serotec, Oxford, UK) with an LOD of 20 pg/ml. Serum testosterone levels were measured using RIA (DPC Coat-A-Count; Diagnostic Products Corp. Los Angeles, CA, USA) with a detection limit of 0.23 nmol/l. Samples with missing values (AMH (boys, n = 1; girls, n = 2), FSH (boys, n = 1; girls, n = 1), Inhibin B (boys, n = 1; girls, n = 6), Testosterone (boys, n = 1), estradiol (girls, n = 1), LH (boys, n = 1; girls, n = 1)) were substituted with the average from the gender and age group.

### Study design

Blood samples (n = 108) obtained at well-defined time points pre- and post-pubertally were selected from our longitudinal cohort. The cohort was not originally designed to include pre- and post-pubertal sampling of DNA and we were therefor only able to include a subset of the complete cohort. However, any child with DNA purified from blood samples before and after pubertal onset was included. These blood samples therefore consisted of paired samples from 32 boys and 22 girls. Clinical data including reproductive hormone values at the pre- and post-pubertal time points where blood sampling for DNA isolation was performed is outlined in Table 1.

### Infinium 450 K array analysis

DNA from the 108 blood samples were purified using standard techniques as described before^6^ and quantified by Qubit (Life Technologies Europe BV, Naerum, Denmark) measurements. The DNA was bisulfite treated, hybridized to Infinium HumanMethylation450 BeadChips (Illumina, San Diego, CA, USA), and scanned using standard protocols by NXT-Dx (Gent, Belgium). Probe intensities were extracted using GenomeStudio V2011.1 (Illumina, San Diego, CA, USA; RRID:SCR_010973). In order to minimize systematic bias samples were randomly distributed onto the BeadChips, which holds 12 samples on each BeadChip. Each BeadChip interrogates 485,577 cytosine positions (CpG sites) in the human genome covering 99% of all RefSeq genes. 365,934 sites are located within known gene regions (promoter, gene body, and untranslated regions) and 119,830 are intergenic sites^46^.

### Data analysis

In GenomeStudio the data was background subtracted and normalized to internal controls. IDAT-files generated by GenomeStudio were imported into the *minfi* (RRID:SCR_012830) package^47^ in RStudio (RRID:SCR_000432) version 0.99.489. The methylation level at each CpG site was calculated as a beta (β) value that ranges from zero (no methylation) to one (complete methylation). The quality of the data was checked using the *ShinyMethyl* package. A principal component analysis (PCA) was performed using the *FactoMineR* package^48^ on the 65 SNPs included on the BeadChip. In the PCA plot the paired samples (pre- and post-pubertal samples) clustered closely together except for three individuals (two girls and one boy) and all six samples were excluded from the subsequent analysis.

Data from the remaining 102 samples from 20 girls and 31 boys were normalized using a Subset quantile Within-Array Normalization (SWAN; RRID:SCR_003455) procedure^49^ and probes containing SNPs in the CpG or extension sites were removed.

It is well known that the cell type composition of blood changes with age and can be a significant confounding factor^50^. Several different algorithms that have been developed to adjust for such confounding effects and we therefore tried to apply two recently developed methods. The *EWASher* algorithm^51^ was applied using Anaconda with Python 2.7 (RRID:SCR_008394) and an improved Surrogate Variable Analysis (SmartSVA, https://github.com/ehsanbehnam/SmartSVA)^52^ was applied in RStudio. With correlation to pubertal age we found that the *EWASher* method reduced the genomic inflation factor from 8.9 to 1.2 (calculated by the *GenABEL* (RRID:SCR_001842) package^53^) but only left very few significant CpGs (Supplementary Figure 5). The SmartSVA method on the other hand both reduced the genomic inflation factor to 1.2 and left 457 significantly associated CpGs at a FDR of 0.05 or less. The SmartSVA method was therefore used in subsequent analyses. The R package *CpGAssoc* (RRID:SCR_000320)^54^ was used to analyse differential methylation at single CpG sites where the results from the modified SVA analysis were included as a covariate. The cpg.assoc function calculates the association of each CpG site to the variable in question and reports effect sizes, standard errors and multiple-testing-adjusted P-values (as well as unadjusted). To account for multiple testing a false discovery rate (FDR) procedure using the Benjamini–Hochberg method^55^ was applied where values below 0.05 were considered significant. In the analysis of pubertal age, chronological age and sex were also included as covariates in the modified SVA and the analysis was performed as a paired analysis.

Differentially methylated regions were investigated using the *DMRcate* package^56^. DMRcate identifies and ranks the most differentially methylated regions across the genome based on tunable kernel smoothing method. A bandwidth of 1000 nucleotides (lambda = 1000) and a scaling factor of 2 (C = 2) were used as recommended by the authors of the *DMRcate* package^56^ and results were corrected for multiple testing by using the Benjamini–Hochberg method^55^. Annotation was done according to hg19.

Heat maps were drawn with the *Heat plus* package in RStudio and venn diagrams and trees were produced with the InteractiVenn web-application^57^.

Gene ontology analysis of differentially methylated CpGs was performed with the *gometh* function within the *missMethyl* package in R. The *gometh* function adapt the *goseq* method of *Young et al.*^58^ and accounts for the number of probes per gene and subsequently performs a modified hypergeometric test. Significant gene ontology terms (p-value < 0.05) were then imported into REVIGO (RRID:SCR_005825) for visualization in a semantic space^59^.

The dataset is available in the ArrayExpress (RRID:SCR_002964) repository ( www.ebi.ac.uk/arrayexpress), under accession number E-MTAB-4187.

### Prediction models

To predict pubertal age and chronological age, the elastic net regression model, which estimated the most predictive of the 467,873 DNA methylation probes, was fitted using the *glmnet* package^60^ in R. The prediction error (standard deviation of residual error from lambda.min) was estimated using 10-fold cross validation. Age at sampling was included as a non-penalized covariate in the regression model to adjust for the age-dependent sampling when pubertal age was analysed. Analysis of biological aging was performed unadjusted.

### Immunohistochemistry

Immunohistochemistry was performed with microwave de-masking in citrate buffer and as described before^61^. Tissue was obtained from the bio bank at the Department of Growth and Reproduction and the primary antibody bought from Abnova (Walnut, CA, USA, Cat. nr. PAB29450) and used in a 1:50 dilution. As negative controls the primary antibody was omitted (Supplementary Figure 4). In total, seven normal adult testicular samples, two pre-pubertal testicular samples, and three ovarian samples were investigated by TRIP6 immunohistochemistry.

### Measurement of circulating TRIP6 levels

A quantitative sandwich ELISA kit (Cat. No. MBS9328439, MyBioSource, Inc., San Diego, CA) was used to measure the concentration of TRIP6 in serum samples from boys (n = 20) and girls (n = 18) at three different time points during pubertal transition. Paired pre-, mid- (around pubertal onset), and post-pubertal samples were from a mean pubertal age of −2.9 (range: −4.8 to −1.6), −0.2 (−0.3 to 0.2) and 3.7 (1.8 to 4.8) in boys and −1.8 (−5.7 to −0.3), 0.1 (−0.5 to 3) and 4.2 (0.9 to 6.2) in girls. The protocol from the manufacturer was followed and the samples were measured as single measurements whereas the standards were measured in duplicates. The manufacturer reports that the inter- and intra-assay CV is less than 15%, the sensitivity is 0.1 ng/ml, the detection rage is 0.625–20 ng/ml and that no cross-reactivity were observed. All samples were within or diluted to fit the detection range. The standard curve was used to convert intensities to concentration and the data analysed by a Wilcoxon Signed Rank test.

## Additional Information

**Accession codes**: ArrayExpress (www.ebi.ac.uk/arrayexpress) accession number E-MTAB-4187.

**How to cite this article**: Almstrup, K. *et al*. Pubertal development in healthy children is mirrored by DNA methylation patterns in peripheral blood. *Sci. Rep.* **6**, 28657; doi: 10.1038/srep28657 (2016).

## Supplementary Material

Supplementary Information

Supplementary Information

## Figures and Tables

**Table 1:** Summary data on the pre- and post-pubertal boys and girls included in the study.

Boys-pre	Age (yrs.)	Pubertal age (yrs.)	Pubic hair (stage)	Testis size (ml)	FSH (IU/l)	LH (IU/l)	AMH (pmol/l)	T (nmol/l)	E2 (pmol/l)	Inhibin B (pg/ml)
Median	9.38	−2.67	1	2	0.64	0.06	600	0.01	1	89
Mean	9.10	−2.47	1	2	0.75	0.10	654	0.018	4	93
1st–3rd Qu	8.98–9.65	−3.07-1.80	1–1	2–2	0.38–0.99	0.04–0.10	506–757	0.01–0.01	1–1	72–109
Range	6.15–10.52	−5.06-0.42	1–2	1–3	0.15–1.75	0.02–0.72	311–1451	0.01–0.17	1–20	40–157
Boys-post										
Median	15.53	3.48	4	18	2.91	2.92	50	14.96	53	204
Mean	15.07	3.50	4	17	3.07	3.04	97	14.53	54	222
1st–3rd Qu	14.58–15.93	3.08–4.42	4–5	15–21	2.36–3.46	2.54–3.35	33–67	12.26–18.11	38–71	167–274
Range	12.57–16.39	0.00–5.95	1–6	4–25	1.09–7.26	1.08–8.51	14–1059	0.42–23.92	1–125	113–362
**Girls-pre**	**Age (yrs.)**	**Pubertal age (yrs.)**	**Pubic hair (stage)**	**Breast (stage)**	**FSH (IU/l)**	** LH (IU/l)**	**AMH (pmol/l)**	**T (nmol/l)**	**E2 (pmol/l)**	**Inhibin B (pg/ml)**
Median	9.06	−1.02	1	1	1.40	0.04	16	0.01	20	19
Mean	8.31	−1.74	1	1	1.70	0.07	20	0.06	23	18
1st–3rd Qu	6.47–9.51	−2.54-0.85	1–1	1–1	1.16–2.09	0.02–0.07	14–25	0.01–0.01	10–26	12–23
Range	5.59–11.32	−6.01-0.42	1–2	1–2	0.08–3.96	0.01–0.50	6–47	0.01–0.60	1–103	3–39
Girls-post										
Median	13.76	3.90	5	5	4.24	3.89	16	0.83	167	46
Mean	14.04	3.99	4	4	4.01	4.00	19	0.93	220	46
1st–3rd Qu	12.59–15.81	3.15–5.53	4–5	4–5	2.56–5.38	2.34–5.51	12–23	0.67–1.19	131–284	24–57
Range	12.20–16.32	0.54–5.95	3–5	3–5	0.73–7.20	0.01–9.41	4–46	0.01–1.98	1–858	8–117

## References

[b1] Sorensen K. . Birth size and age at menarche: a twin perspective. Hum Reprod 28, 2865–2871, 10.1093/humrep/det283 (2013). 23925395

[b2] Abreu A. P. . Central Precocious Puberty Caused by Mutations in the Imprinted Gene MKRN3. New England Journal of Medicine 368, 2467–2475, 10.1056/NEJMoa1302160 (2013). 23738509 PMC3808195

[b3] Boehm U. . Expert consensus document: European Consensus Statement on congenital hypogonadotropic hypogonadism–pathogenesis, diagnosis and treatment. Nature reviews. Endocrinology 11, 547–564, 10.1038/nrendo.2015.112 (2015). 26194704

[b4] Perry J. R. . Parent-of-origin-specific allelic associations among 106 genomic loci for age at menarche. Nature 514, 92–97, 10.1038/nature13545 (2014). 25231870 PMC4185210

[b5] Elks C. E. . Thirty new loci for age at menarche identified by a meta-analysis of genome-wide association studies. Nat Genet 42, 1077–1085, 10.1038/ng.714 (2010). 21102462 PMC3140055

[b6] Hagen C. P. . Pubertal onset in girls is strongly influenced by genetic variation affecting FSH action. Scientific reports 4, 6412, 10.1038/srep06412 (2014). 25231187 PMC4166707

[b7] Hannum G. . Genome-wide methylation profiles reveal quantitative views of human aging rates. Molecular cell 49, 359–367, 10.1016/j.molcel.2012.10.016 (2013). 23177740 PMC3780611

[b8] Horvath S. DNA methylation age of human tissues and cell types. Genome biology 14, R115, 10.1186/gb-2013-14-10-r115 (2013). 24138928 PMC4015143

[b9] Bekaert B., Kamalandua A., Zapico S. C., Van de Voorde W. & Decorte R. Improved age determination of blood and teeth samples using a selected set of DNA methylation markers. Epigenetics , 1–9, 10.1080/15592294.2015.1080413 (2015). PMC4844214 26280308

[b10] Bell J. T. . Epigenome-wide scans identify differentially methylated regions for age and age-related phenotypes in a healthy ageing population. PLoS Genet 8, e1002629, 10.1371/journal.pgen.1002629 (2012). 22532803 PMC3330116

[b11] Marioni R. E. . DNA methylation age of blood predicts all-cause mortality in later life. Genome biology 16, 25, 10.1186/s13059-015-0584-6 (2015). 25633388 PMC4350614

[b12] Weidner C. I. . Aging of blood can be tracked by DNA methylation changes at just three CpG sites. Genome biology 15, R24, 10.1186/gb-2014-15-2-r24 (2014). 24490752 PMC4053864

[b13] Richmond R. C. . Prenatal exposure to maternal smoking and offspring DNA methylation across the lifecourse: findings from the Avon Longitudinal Study of Parents and Children (ALSPAC). Hum Mol Genet 24, 2201–2217, 10.1093/hmg/ddu739 (2015). 25552657 PMC4380069

[b14] Zhang Y. . Smoking-Associated DNA Methylation Biomarkers and Their Predictive Value for All-Cause and Cardiovascular Mortality. Environ Health Perspect , 10.1289/ehp.1409020 (2015). PMC4710597 26017925

[b15] Aslibekyan S. . Epigenome-wide study identifies novel methylation loci associated with body mass index and waist circumference. Obesity (Silver Spring, Md.) 23, 1493–1501, 10.1002/oby.21111 (2015). PMC4482015 26110892

[b16] Horvath S. . Obesity accelerates epigenetic aging of human liver. Proc Natl Acad Sci USA 111, 15538–15543, 10.1073/pnas.1412759111 (2014). 25313081 PMC4217403

[b17] Heijmans B. T. . Persistent epigenetic differences associated with prenatal exposure to famine in humans. Proc Natl Acad Sci USA 105, 17046–17049, 10.1073/pnas.0806560105 (2008). 18955703 PMC2579375

[b18] Tobi E. W. . DNA methylation signatures link prenatal famine exposure to growth and metabolism. Nature communications 5, 5592, 10.1038/ncomms6592 (2014). PMC4246417 25424739

[b19] Demetriou C. A. . Methylome analysis and epigenetic changes associated with menarcheal age. PLoS One 8, e79391, 10.1371/journal.pone.0079391 (2013). 24278132 PMC3835804

[b20] Lunetta K. L. . Rare coding variants and X-linked loci associated with age at menarche. Nature communications 6, 7756, 10.1038/ncomms8756 (2015). PMC4538850 26239645

[b21] Lee J. W., Choi H. S., Gyuris J., Brent R. & Moore D. D. Two classes of proteins dependent on either the presence or absence of thyroid hormone for interaction with the thyroid hormone receptor. Molecular endocrinology 9, 243–254, 10.1210/mend.9.2.7776974 (1995). 7776974

[b22] Lv K. . Trip6 promotes dendritic morphogenesis through dephosphorylated GRIP1-dependent myosin VI and F-actin organization. J Neurosci 35, 2559–2571, 10.1523/JNEUROSCI.2125-14.2015 (2015). 25673849 PMC6605620

[b23] Lin F.-T., Lin V. Y., Lin V. T. G. & Lin W.-C. TRIP6 antagonizes the recruitment of A20 and CYLD to TRAF6 to promote the LPA2 receptor-mediated TRAF6 activation. Cell Discovery 2, 15048 (2016). 27134758 10.1038/celldisc.2015.48 PMC4850058

[b24] Diefenbacher M. E., Litfin M., Herrlich P. & Kassel O. The nuclear isoform of the LIM domain protein Trip6 integrates activating and repressing signals at the promoter-bound glucocorticoid receptor. Molecular and Cellular Endocrinology 320, 58–66 (2010). 20153803 10.1016/j.mce.2010.02.010

[b25] McBryan J., Howlin J., Kenny P. A., Shioda T. & Martin F. ERalpha-CITED1 co-regulated genes expressed during pubertal mammary gland development: implications for breast cancer prognosis. Oncogene 26, 6406–6419, 10.1038/sj.onc.1210468 (2007). 17486082

[b26] Ron M. . Combining mouse mammary gland gene expression and comparative mapping for the identification of candidate genes for QTL of milk production traits in cattle. BMC Genomics 8, 183, 10.1186/1471-2164-8-183 (2007). 17584498 PMC1906769

[b27] Tsutsumi M. . Screening of genes involved in chromosome segregation during meiosis I: *in vitro* gene transfer to mouse fetal oocytes. J Hum Genet 57, 515–522, 10.1038/jhg.2012.61 (2012). 22648182

[b28] Parent A. S. . Gene expression profiling of hypothalamic hamartomas: a search for genes associated with central precocious puberty. Horm Res 69, 114–123, 10.1159/000111815 (2008). 18059092

[b29] Arambepola N. K., Bunick D. & Cooke P. S. Thyroid hormone effects on androgen receptor messenger RNA expression in rat Sertoli and peritubular cells. J Endocrinol 156, 43–50 (1998). 9496232 10.1677/joe.0.1560043

[b30] Manna P. R. . Assessment of mechanisms of thyroid hormone action in mouse Leydig cells: regulation of the steroidogenic acute regulatory protein, steroidogenesis, and luteinizing hormone receptor function. Endocrinology 142, 319–331, 10.1210/endo.142.1.7900 (2001). 11145595

[b31] Zhang C. . Effects of 3, 5, 3′-triiodothyronine (t3) and follicle stimulating hormone on apoptosis and proliferation of rat ovarian granulosa cells. The Chinese journal of physiology 56, 298–305, 10.4077/cjp.2013.bab186 (2013). 24032715

[b32] Zhang C., Xia G. & Tsang B. K. Interactions of thyroid hormone and FSH in the regulation of rat granulosa cell apoptosis. Frontiers in bioscience (Elite edition) 3, 1401–1413 (2011). 21622145 10.2741/E342

[b33] Vervenne H. B. . Targeted disruption of the mouse Lipoma Preferred Partner gene. Biochemical and biophysical research communications 379, 368–373, 10.1016/j.bbrc.2008.12.074 (2009). 19111675

[b34] Hoffman L. M. . Genetic ablation of zyxin causes Mena/VASP mislocalization, increased motility, and deficits in actin remodeling. J Cell Biol 172, 771–782, 10.1083/jcb.200512115 (2006). 16505170 PMC2063708

[b35] Renfranz P. J., Blankman E. & Beckerle M. C. The cytoskeletal regulator zyxin is required for viability in Drosophila melanogaster. Anat Rec (Hoboken) 293, 1455–1469, 10.1002/ar.21193 (2010). 20648572 PMC2939194

[b36] Leicher T., Bahring R., Isbrandt D. & Pongs O. Coexpression of the KCNA3B gene product with Kv1.5 leads to a novel A-type potassium channel. J Biol Chem 273, 35095–35101 (1998). 9857044 10.1074/jbc.273.52.35095

[b37] Miller J. A. . Conserved molecular signatures of neurogenesis in the hippocampal subgranular zone of rodents and primates. Development 140, 4633–4644 (2013). 24154525 10.1242/dev.097212 PMC3817946

[b38] Xu J. . The voltage-gated potassium channel Kv1.3 regulates energy homeostasis and body weight. Human Molecular Genetics 12, 551–559, 10.1093/hmg/ddg049 (2003). 12588802

[b39] Yousefi P. . Sex differences in DNA methylation assessed by 450 K BeadChip in newborns. BMC Genomics 16, 911, 10.1186/s12864-015-2034-y (2015). 26553366 PMC4640166

[b40] Finucane H. K. . Partitioning heritability by functional annotation using genome-wide association summary statistics. Nat Genet 47, 1228–1235, 10.1038/ng.3404 (2015). 26414678 PMC4626285

[b41] Heyn H. . Distinct DNA methylomes of newborns and centenarians. Proc Natl Acad Sci USA 109, 10522–10527, 10.1073/pnas.1120658109 (2012). 22689993 PMC3387108

[b42] Aksglaede L., Sorensen K., Petersen J. H., Skakkebaek N. E. & Juul A. Recent decline in age at breast development: the Copenhagen Puberty Study. Pediatrics 123, e932–939, 10.1542/peds.2008-2491 (2009). 19403485

[b43] Hagen C. P. . Individual serum levels of anti-Mullerian hormone in healthy girls persist through childhood and adolescence: a longitudinal cohort study. Hum Reprod 27, 861–866, 10.1093/humrep/der435 (2012). 22215627

[b44] Sorensen K., Aksglaede L., Petersen J. H. & Juul A. Recent changes in pubertal timing in healthy Danish boys: associations with body mass index. The Journal of clinical endocrinology and metabolism 95, 263–270, 10.1210/jc.2009-1478 (2010). 19926714

[b45] Marshall W. A. & Tanner J. M. Variations in pattern of pubertal changes in girls. Archives of disease in childhood 44, 291–303 (1969). 5785179 10.1136/adc.44.235.291 PMC2020314

[b46] Sandoval J. . Validation of a DNA methylation microarray for 450,000 CpG sites in the human genome. Epigenetics 6, 692–702 (2011). 21593595 10.4161/epi.6.6.16196

[b47] Aryee M. J. . Minfi: a flexible and comprehensive Bioconductor package for the analysis of Infinium DNA methylation microarrays. Bioinformatics 30, 1363–1369, 10.1093/bioinformatics/btu049 (2014). 24478339 PMC4016708

[b48] Le S., Josse J. & Husson F. FactoMineR: An R package for multivariate analysis. J Stat Softw 25, 1–18 (2008).

[b49] Maksimovic J., Gordon L. & Oshlack A. SWAN: Subset-quantile within array normalization for illumina infinium HumanMethylation450 BeadChips. Genome biology 13, R44, 10.1186/gb-2012-13-6-r44 (2012). 22703947 PMC3446316

[b50] Jaffe A. E. & Irizarry R. A. Accounting for cellular heterogeneity is critical in epigenome-wide association studies. Genome biology 15, R31, 10.1186/gb-2014-15-2-r31 (2014). 24495553 PMC4053810

[b51] Zou J., Lippert C., Heckerman D., Aryee M. & Listgarten J. Epigenome-wide association studies without the need for cell-type composition. Nature methods 11, 309–311, 10.1038/nmeth.2815 (2014). 24464286

[b52] Leek J. T. & Storey J. D. Capturing heterogeneity in gene expression studies by surrogate variable analysis. PLoS Genet 3, 1724–1735, 10.1371/journal.pgen.0030161 (2007). 17907809 PMC1994707

[b53] Aulchenko Y. S., Ripke S., Isaacs A. & van Duijn C. M. GenABEL: an R library for genome-wide association analysis. Bioinformatics 23, 1294–1296, 10.1093/bioinformatics/btm108 (2007). 17384015

[b54] Barfield R. T., Kilaru V., Smith A. K. & Conneely K. N. CpGassoc: an R function for analysis of DNA methylation microarray data. Bioinformatics 28, 1280–1281, 10.1093/bioinformatics/bts124 (2012). 22451269 PMC3577110

[b55] Benjamini Y., Drai D., Elmer G., Kafkafi N. & Golani I. Controlling the false discovery rate in behavior genetics research. Behav Brain Res 125, 279–284 (2001). 11682119 10.1016/s0166-4328(01)00297-2

[b56] Peters T. J. . De novo identification of differentially methylated regions in the human genome. Epigenetics & chromatin 8, 6, 10.1186/1756-8935-8-6 (2015). 25972926 PMC4429355

[b57] Heberle H., Meirelles G. V., da Silva F. R., Telles G. P. & Minghim R. InteractiVenn: a web-based tool for the analysis of sets through Venn diagrams. BMC bioinformatics 16, 169, 10.1186/s12859-015-0611-3 (2015). 25994840 PMC4455604

[b58] Young M. D., Wakefield M. J., Smyth G. K. & Oshlack A. Gene ontology analysis for RNA-seq: accounting for selection bias. Genome biology 11, R14, 10.1186/gb-2010-11-2-r14 (2010). 20132535 PMC2872874

[b59] Supek F., Bosnjak M., Skunca N. & Smuc T. REVIGO summarizes and visualizes long lists of gene ontology terms. PLoS One 6, e21800, 10.1371/journal.pone.0021800 (2011). 21789182 PMC3138752

[b60] Friedman J., Hastie T. & Tibshirani R. Regularization Paths for Generalized Linear Models via Coordinate Descent. J Stat Softw 33, 1–22 (2010). 20808728 PMC2929880

[b61] Blomberg Jensen M. . Vitamin D receptor and vitamin D metabolizing enzymes are expressed in the human male reproductive tract. Hum Reprod 25, 1303–1311, 10.1093/humrep/deq024 (2010). 20172873

