# Erratum: Pubertal development in healthy children is mirrored by DNA methylation patterns in peripheral blood

**DOI:** 10.1038/srep30664

**Published:** 2016-08-03

**Authors:** Kristian Almstrup, Marie Lindhardt Johansen, Alexander S. Busch, Casper P. Hagen, John E. Nielsen, Jørgen Holm Petersen, Anders Juul

Scientific Reports 6: Article number: 28657; 10.1038/srep28657 published online: 06 28 2016; updated: 08 03 2016.

This Article contains typographical errors in Table 1. In the column ‘Pubertal age’ the values for Boys-pre ‘1^st^–3^rd^ Qu’ and ‘Range’ “−3.07–1.80” and “−5.06–0.42” should read: “of “−3.07– −1.80” and “−5.06– −0.42” respectively. In addition, the Girls-pre values for ‘1^st^–3^rd^ Qu’ and ‘Range’ “−2.54–0.85” and “−6.01–0.42” should read: “−2.54– −0.85” and “−6.01– −0.42” respectively.

