# Supplementary material for: Pubertal development in healthy children is mirrored by DNA methylation patterns in peripheral blood
Source: Sci Rep. 2016 Jun 28;6:28657. doi: 10.1038/srep28657 (PMC4923870; doi:10.1038/srep28657)

Supplementary figures

Pubertal development in healthy children is mirrored by DNA methylation patterns in peripheral blood. Kristian Almstrup, Marie Lindhardt Johansen, Alexander S. Busch, Casper P. Hagen, John E. Nielsen, Jørgen Holm Petersen and Anders Juul.

Supplementary figure 1

DNA methylation levels in defined genomic regions analysed according to time of sampling (pre- and post-pubertal) and sex. A Wilcoxon rank sum test was used to test for differences. N=North, S=South, NS=Non Significant, *=P-value below 0.05, **=P-value below 0.01.

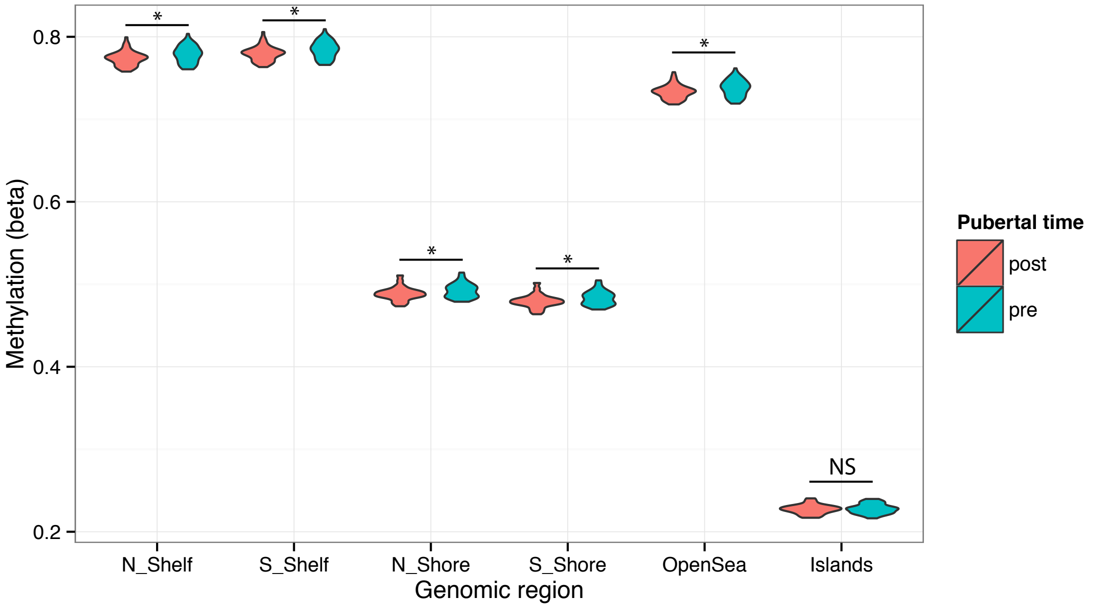

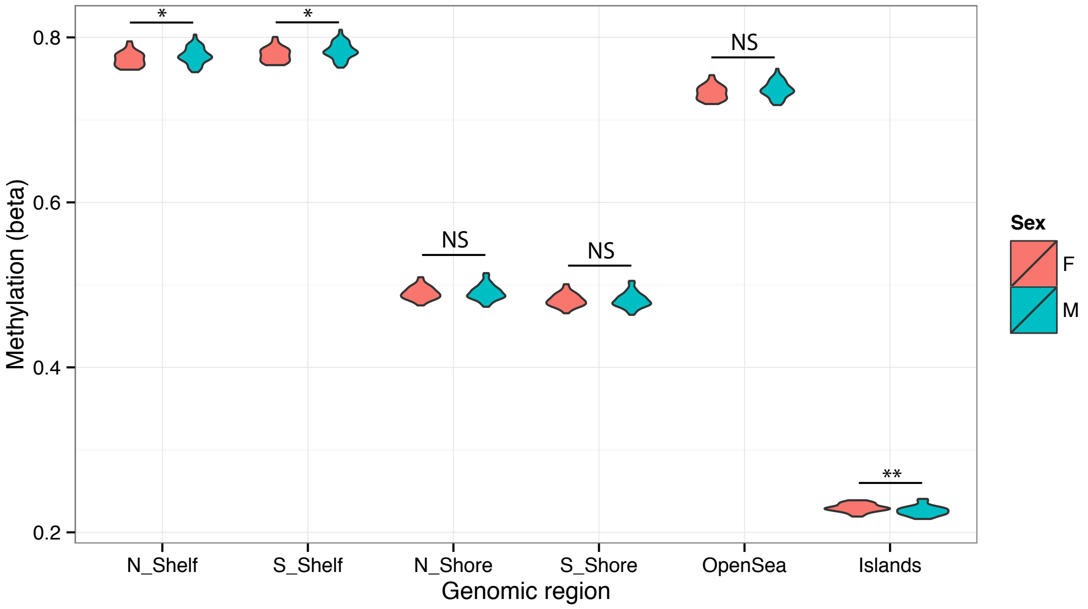

Supplementary figure 2

Unsupervised hierarchical clustering of the unique set of 133 CpGs that correlate with circulating reproductive hormones among boys.

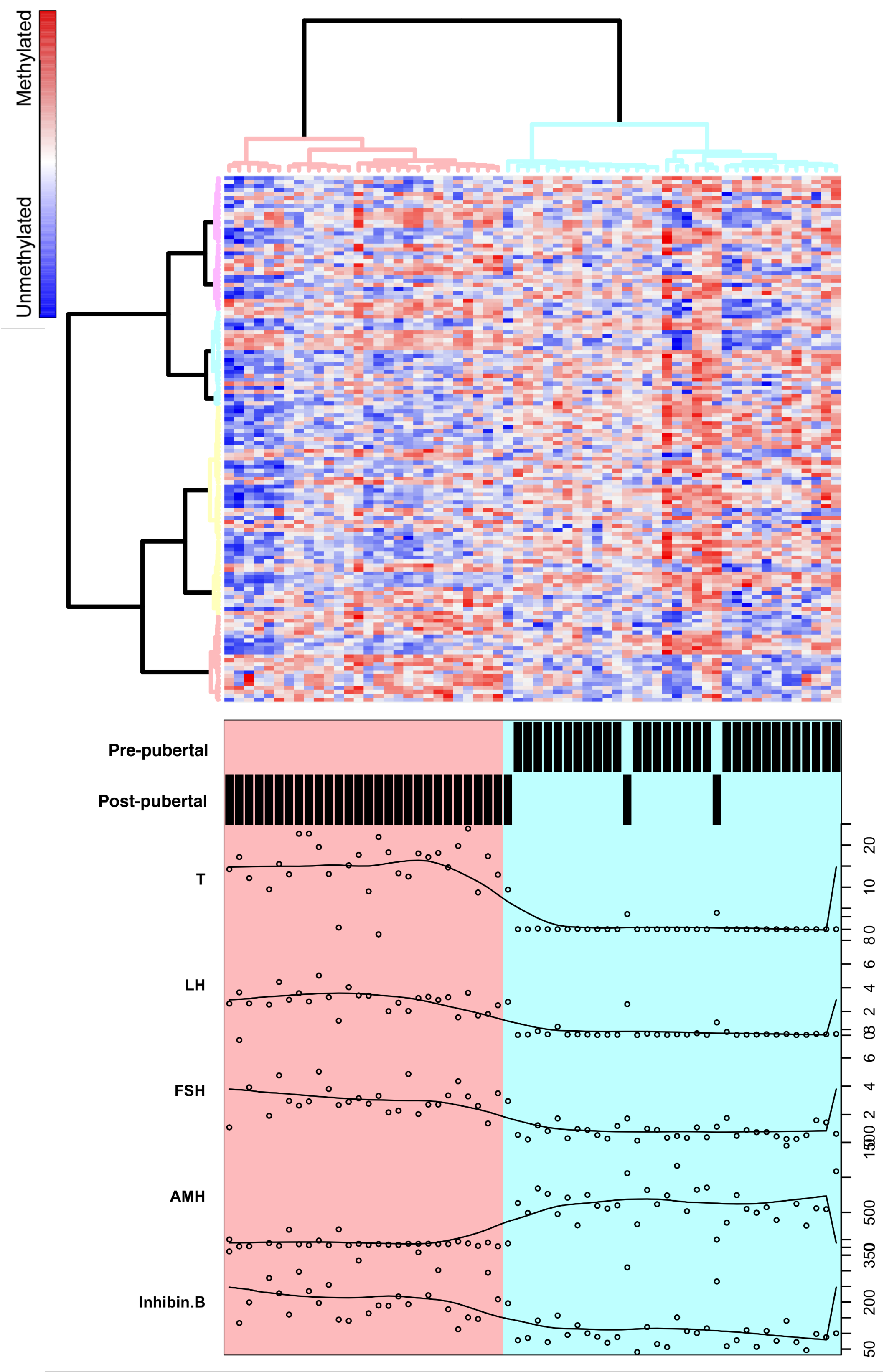

Supplementary figure 3

Region on chromosome 7 situated between *SLC12A9* and *TRIP6* (hg19 coordinates chr7:100463206-100464771) that co-ordinately change in DNA methylation during puberty for boys and girls individually.

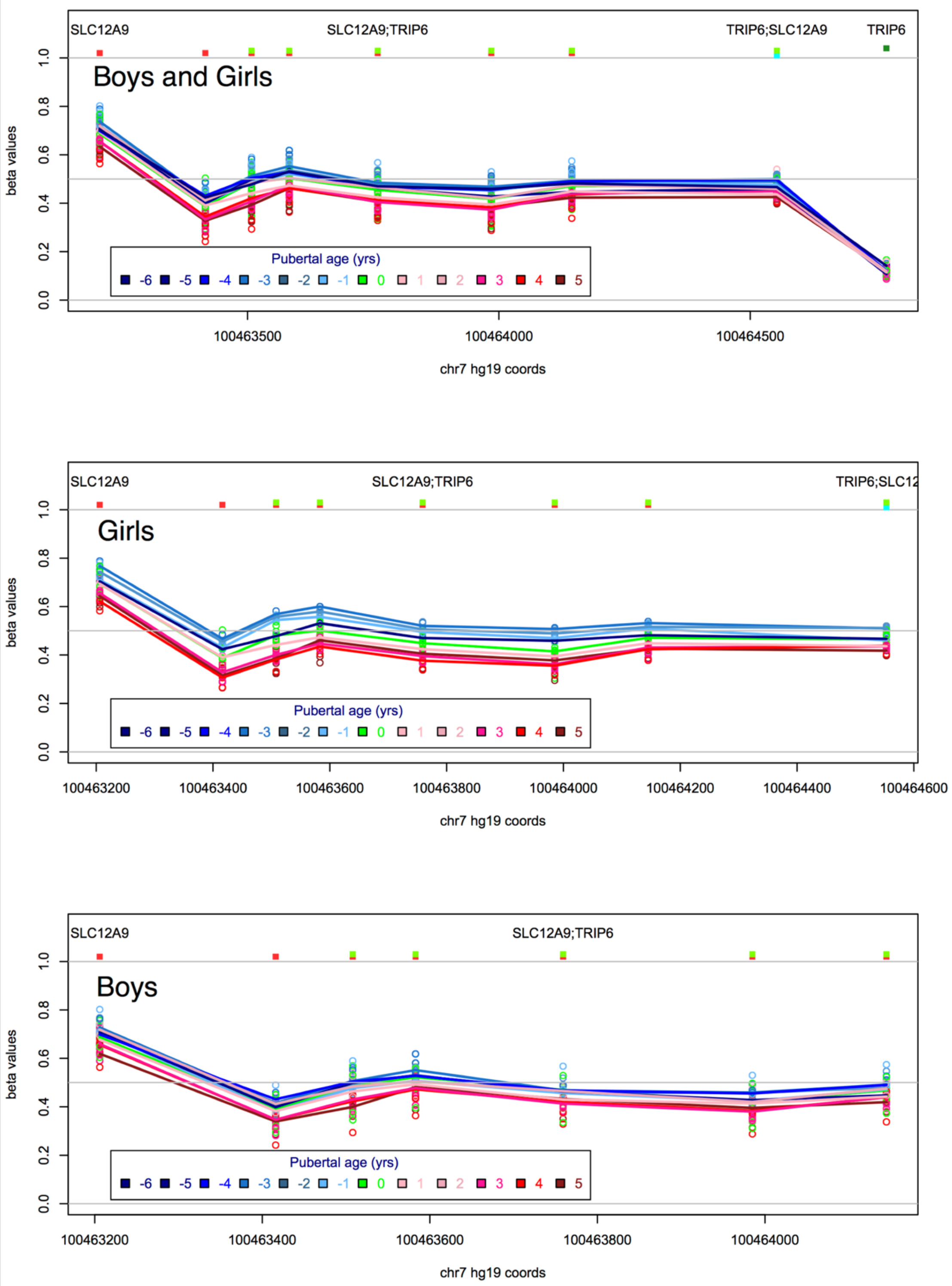

Supplementary figure 4

Immunohistochemical staining for TRIP6 in testicular tissues from pre and post-pubertal samples (similar to figure 3B and C) together with negative controls (omission of primary antibody) on serial sections. In addition, to the description in the main text, a faint and variable staining was observed in spermatocytes and round spermatids. TRIP6 staining on ovarian tissue (16 years old girl) showing a weak staining in oocytes and granulosa cells, whereas the theca cells (marked by CYP11A1) are negative for TRIP6 (from 11 years old girl).

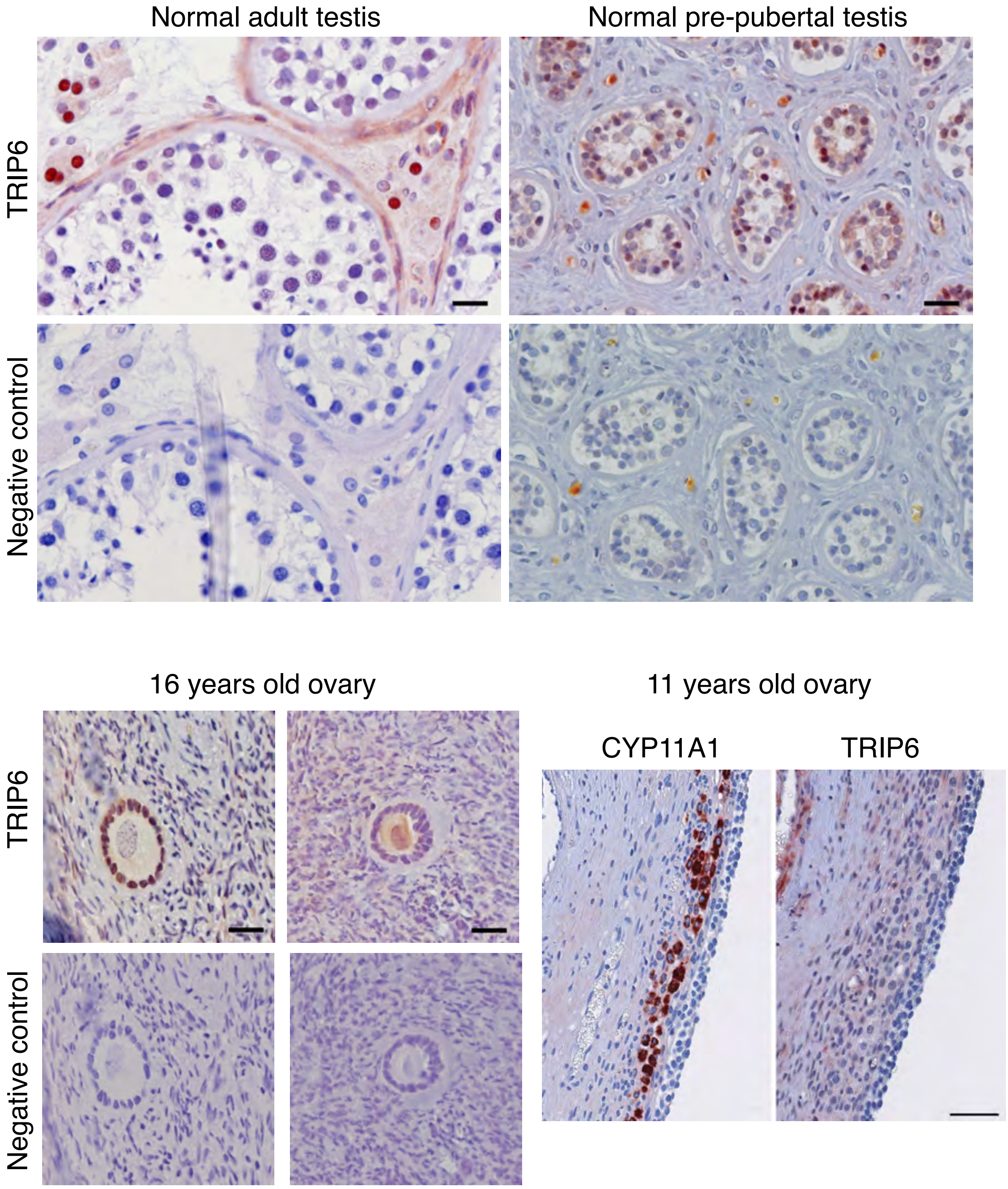

Supplementary figure 5

Examples of qq-plots and calculated genomic inflation factors using the unadjusted, *EWASher* or the modified surrogate variant analysis (SVA).

Unadjusted P-values, genomic inflation factor 8.9 (SE 0.072).

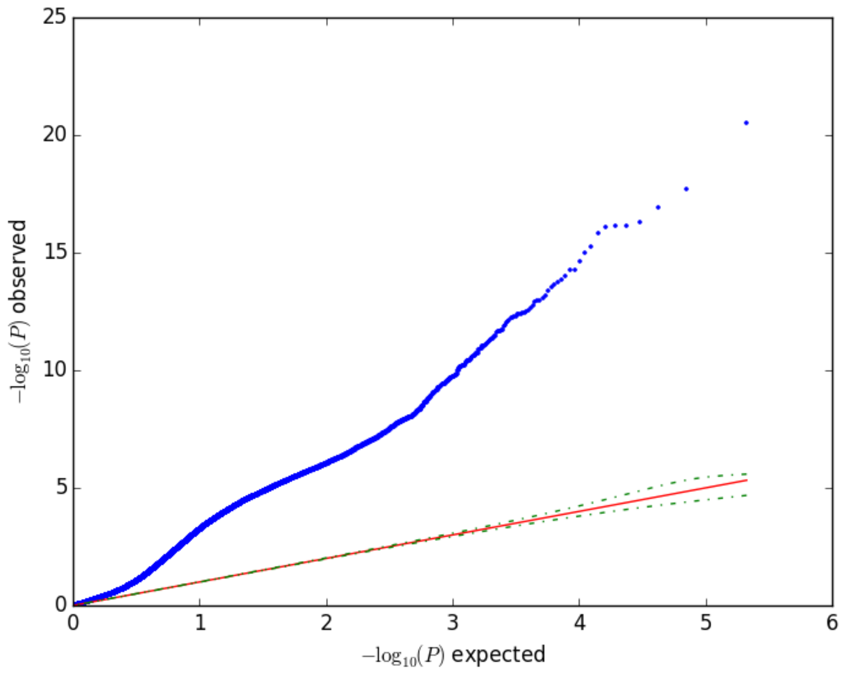

EWASher adjusted, genomic inflation factor 1.2 (SE 0.0002)

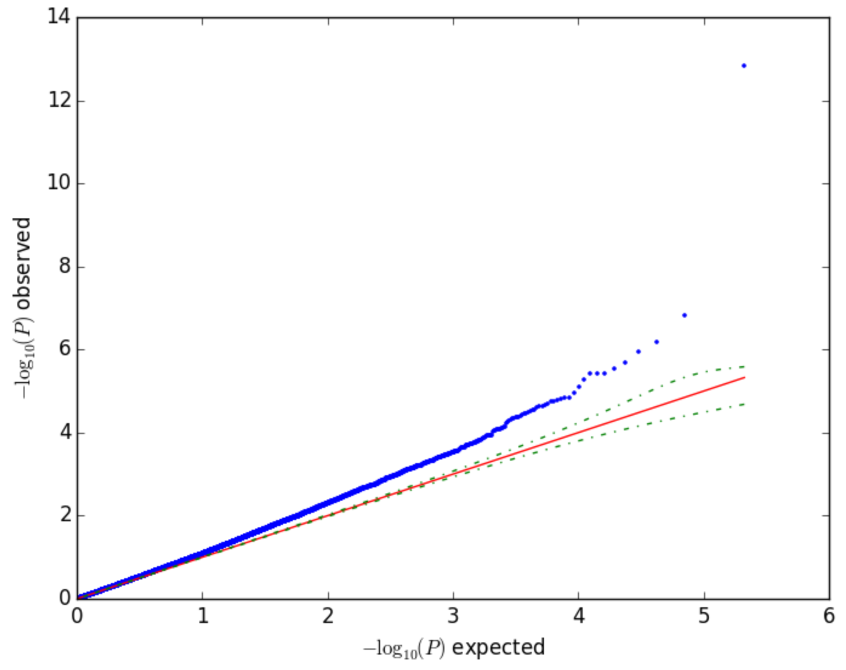

SVA adjusted, genomic inflation factor 1.2 (SE 0.0002)

(age included as covariate and paired analysis)

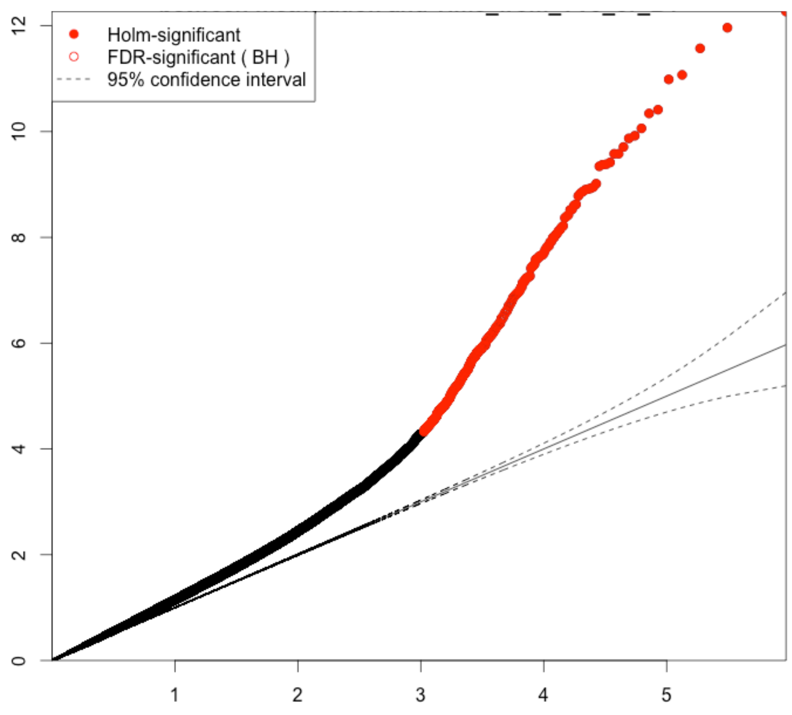

Supplement: Supplementary Information [file srep28657-s1.pdf]
